# CRISPR/Cas13-Based Approaches for Ultrasensitive and Specific Detection of microRNAs

**DOI:** 10.3390/cells10071655

**Published:** 2021-07-01

**Authors:** Javier T. Granados-Riveron, Guillermo Aquino-Jarquin

**Affiliations:** 1 Laboratorio de Investigación en Patogénesis Molecular, Hospital Infantil de México, Federico Gómez, Ciudad de México 06720, Mexico; javiertgranados@himfg.edu.mx; 2 Laboratorio de Investigación en Genómica, Genética y Bioinformática, Hospital Infantil de México, Federico Gómez, Ciudad de México 06720, Mexico

**Keywords:** CRISPR-Cas13 technology, miRNA detection, biosensing, signal amplification

## Abstract

MicroRNAs (miRNAs) have a prominent role in virtually every aspect of cell biology. Due to the small size of mature miRNAs, the high degree of similarity between miRNA family members, and the low abundance of miRNAs in body fluids, miRNA expression profiling is technically challenging. Biosensors based on electrochemical detection for nucleic acids are a novel category of inexpensive and very sensitive diagnostic tools. On the other hand, after recognizing the target sequence, specific CRISPR-associated proteins, including orthologues of Cas12, Cas13, and Cas14, exhibit collateral nonspecific catalytic activities that can be employed for specific and ultrasensitive nucleic acid detection from clinically relevant samples. Recently, several platforms have been developed, connecting the benefits of enzyme-assisted signal amplification and enzyme-free amplification biosensing technologies with CRISPR-based approaches for miRNA detection. Together, they provide high sensitivity, precision, and fewer limitations in diagnosis through efficient sensors at a low cost and a simple miniaturized readout. This review provides an overview of several CRISPR-based biosensing platforms that have been developed and successfully applied for ultrasensitive and specific miRNA detection.

## 1. Introduction

MicroRNAs (miRNA) include a set of short, noncoding proteins and small RNA molecules with a length of 20–24 nt, generated by the RNase-III-type enzyme Dicer from an endogenous transcript that contains a local hairpin structure [1,2]. After unwinding, the miRNA forms part of the RNA-induced silencing complex (RISC) assembly and causes translational repression or mRNA degradation [3].

The primary signature of miRNAs is their master role in repressing the expression of multiple protein-coding genes at the transcriptional, post-transcriptional, and translational levels by binding to the promoter regions, 3′ untranslated region (3′-UTR), 5′-UTR, and the coding regions of target sequences [3,4,5].

Recently, the universality of miRNAs in research has continuously grown, as the presence or dysregulation of distinct miRNAs can indicate specific pathologic conditions. In some cases, several miRNAs have been identified as oncogenic elements or as tumor suppressors, and in other cases, they have been shown to participate in diverse pathways, having different effects on cell survival, growth, and proliferation depending on the cell type and the pattern of gene expression [6,7]. For example, miR-17 has been selected as a proof-of-concept model target for different biosensors because of its role in many diseases, including breast cancer [8], pulmonary hypertension [9], and Mantle cell lymphoma [10]. Therefore, miRNAs offer new possibilities for potential diagnostic and prognostic tools in miRNA-associated diseases such as cancer [4,7]. Considering the master role that miRNAs play in regulating critical biological processes and that their ability to repress their targets crucially depends on their expression levels [3,5,11], it is highly desirable to construct specific and ultrasensitive methods for the detection of miRNAs showing low expression levels.

## 2. Molecular Mechanisms of RNA Targeting by CRISPR-Cas13

**Figure 1:**
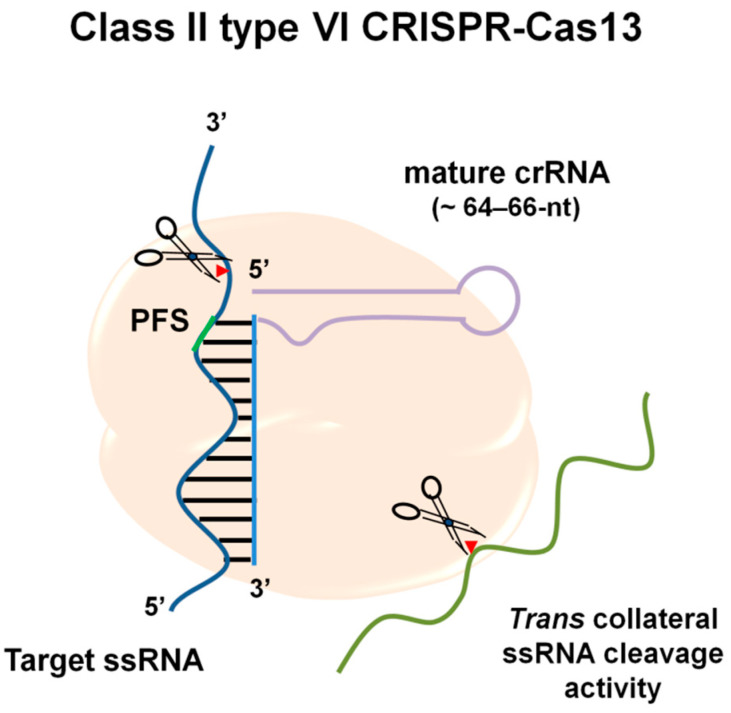
*Mechanism of action of CRISPR-Cas13.* The simplicity of the CRISPR-Cas13 system comprises two components: the programmable single-effector RNA-guided RNase Cas13 and a 64–66 nt CRISPR RNA (crRNA), best known as guide RNA, which recognizes the target RNA through the PFS (green), analogous to the protospacer adjacent motif, or PAM, a sequence recognized by Cas9. Scissors represent *cis* (on target) and *trans* cleavage (promiscuous cleavage), exerted by Cas13 through two HEPN domains, which together form the active ribonuclease site. This depiction represents the targeting or viral interference stage in the ‘adaptive’ immune system in bacteria and archaea, where foreign genetic elements or target ssRNA (e.g., the invading phage ssRNA) is cleaved by the crRNA–Cas13 complex. PFS: Protospacer-flanking site.

Class II type VI CRISPR-Cas systems are RNA-guided and RNA-targeting machineries that provide prokaryote immunity against RNA elements, such as RNA phages [12,13]. All type VI CRISPR-Cas systems have a single effector protein known as the Cas13 effector, previously known as the C2c2 CRISPR-Cas system [12,14]. To date, four Cas13 protein families have been identified, including Cas13a, Cas13b, Cas13c, and Cas13d [15,16,17]. Cas13 effectors possess an RNase activity provided by the two higher eukaryotic and prokaryotic nucleotide-binding domains (HEPN), which is required for target RNA degradation and the processing and maturation of the pre-CRISPR RNA (crRNA) into mature crRNA (64–66-nt in length) to ensure target specificity [14,18]. By recognizing a short hairpin in the crRNA, the Cas13 protein forms complexes with the guide RNA. Target specificity is determined by a 28–30 nt spacer complementary to the target region via the protospacer-flanking site (PFS) sequence (Figure 1).

Once activated through the specific binding to the crRNA-complementary ssRNA target, the Cas13 effectors promote cis cleavage activity, where the target RNA is cleaved by the crRNA–Cas13 complex. At the same time, Cas13 effectors exert a nonspecific cleavage of collateral ssRNAs in trans (also termed collateral “promiscuous” activity) [12,14] (Figure 1). This collateral activity has been harnessed to develop highly sensitive pathogen detection methods [19,20].

In addition, to expand the usefulness of Cas13 as a tool for studying RNA, it is possible to create a catalytically dead variant (dCas13) by mutating catalytic arginine residues within the two conserved HEPN domains. Thus, catalytically inactive Cas13a maintains targeted RNA binding, allowing for programmable tracking of transcripts in live cells [19].

Furthermore, Cas12, Cas13, and Cas14a have also been reported to have a target-activated *trans*-cleavage activity that can efficiently cleave ssDNA or ssRNA sequences, making them suitable for the detection of signal-amplified nucleic acids [20,21,22].

In Table 1, relevant information regarding the main applications of CRISPR-Cas12, -Cas13, and -Cas14 and the significant differences between them are summarized.

Particularly for Cas13, experimental characterization of this effector protein reveals a highly versatile resource for new RNA-targeting technologies that have been recently developed for RNA knockdown [17,19,25], editing [25], splicing, viral delivery [17] in mammalian cells, and, potentially, miRNA detection.

This review paper provides an overview of current approaches employing CRISPR-Cas13-based biosensor systems, highlighting how they have allowed the miniaturization of electrochemical transducers, improving their sensitivity, specificity, and applicability for miRNA diagnosis.

## 3. Nucleic Acid Amplification Technologies for miRNA Detection

### 3.1. qPCR: The Standard Gold Method

Traditional methods of studying miRNA levels include microarrays, RNA sequencing methods, and the quantitative polymerase chain reaction (qPCR), the current gold standard method [28,29,30]. Although these methods are effective, they require qualified personnel to run the tests and interpret the results. Moreover, their implementation requires the standardization of protocols and expensive equipment, hindering their use in field-deployable diagnostics [31].

In terms of the “gold standard” method, exact quantification by real-time qPCR relies on optimizing multiple steps, such as RNA extraction, RNA integrity control, cDNA synthesis, primer design, amplicon detection, and data normalization, to obtain meaningful and reproducible results [29,32].

In a two-step real-time qPCR reaction, the target miRNA is first retrotranscribed by stem-loop real-time primers, poly(T) adapter, or a gene-specific primer that includes a tail sequence [33,34]. Subsequently, the reverse transcripts are amplified using the TaqMan probe method or SYBR Green fluorescent dye method assay to achieve real-time fluorescence detection [34]. The absolute quantity can be determined by creating a standard curve with a molecule of known concentration and comparing this to samples of interest at unknown concentrations. Alternatively, the relative quantification method allows the comparison of target RNA molecules, which may vary in abundance between different tissue types or as a result of different treatments in respect to a reference RNA target(s) whose abundance does not vary significantly. Concentrations in qPCR are expressed in the logarithmic scale [35].

### 3.2. Isothermal Nucleic Acid Amplification Technologies

Recently, isothermal nucleic acid amplification technologies, including enzyme-assisted amplification methods (such as rolling circle amplification (RCA), strand displacement amplification (SDA), and loop-mediated isothermal amplification (LAMP)) and enzyme-free amplification methods (including hybridization chain reaction (HCR), DNAzyme, and entropy-driven circuit (EDC), and catalytic hairpin assembly (CHA)), have been employed to improve the sensitivity and accuracy of miRNA detection [29,36,37]. However, the enzyme-assisted amplification methods, for example, have the shortcomings of high cost, burdensome storage requirements, and complicated reaction conditions [29,36,37].

More recently, novel CRISPR-based nucleic-acid detection platforms have emerged, attracting keen attention as rapid and sensitive methods for miRNA detection and overcoming important limitations of the RT-qPCR method and other nucleic acid amplification technologies [31,38].

## 4. Engineering RNA Sensors Utilizing the CRISPR-Cas13 Machinery

The CRISPR-Cas13a system has significantly low off-target effects. Its activity is severely reduced or abrogated by 1–2 mismatches in the center of a guide RNA (gRNA), allowing for a mismatch control for each targeting gRNA [19,39]. In this regard, the CRISPR-Cas13a system has already been used as a rapid DNA or RNA detection method with attomolar sensitivity and single-base mismatch specificity through the detection system SHERLOCK (specific high-sensitivity enzymatic reporter unlocking), developed by Gootenberg et al. [26].

SHERLOCK is a Cas13-based nucleic acid detection technology that uses the isothermal recombinase polymerase amplification (RPA) followed by in vitro transcription and Cas13 detection [26]. The specificity of Cas13 is conferred by crRNA–target pairing, and further sensitivity is achieved through signal amplification by Cas13’s collateral cleavage activity on the RNA reporters added to the reaction. The signal can be captured on a colorimetric lateral-flow strip (by biotin–fluorescein RNA reporters) or visualized by fluorescence signal readout (by molecular beacon fluorescent reporters) to enable the rapid detection of almost any kind of suitable Cas13 RNA target. In this way, SHERLOCK allows rapid (in as little as one hour with a setup time shorter than 15 min) and reliable nucleic acid detection at concentrations down to approximately 2 aM (10^−18^ molar) and single base-pair mismatch specificity [40].

In this regard, sensitive and accurate tools for whole or partial miRNome detection and quantification are within reach, for example, to diagnose or assess the severity of miRNA-associated diseases. However, although the use of SHERLOCK as such has not been specifically reported for miRNA detection, this platform laid the foundation for next-generation CRISPR-based diagnostics employing the Cas13a protein effector [41], as will be reviewed in the following sections.

## 5. Fluorescent miRNA Detection Based on CRISPR-Cas13

### 5.1. Single-Step Cas13a-Triggered Signal Amplification System for miR-17

**Figure 2:**
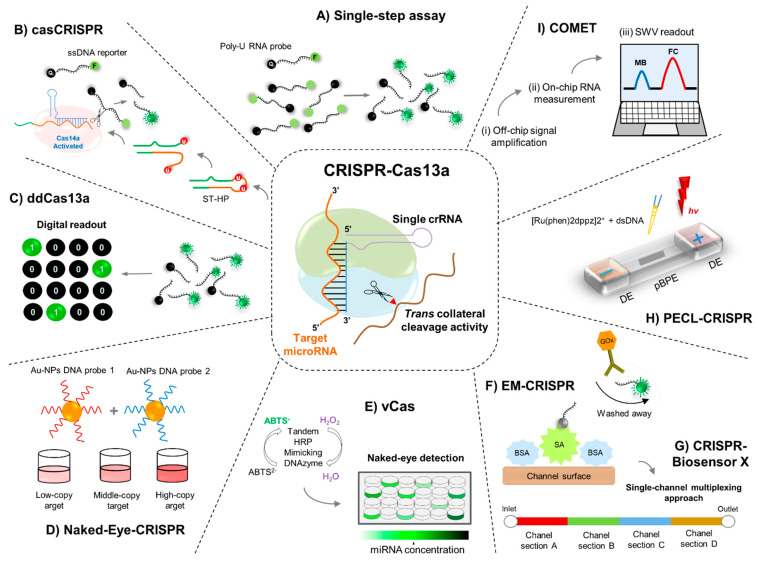
(i) *Fluorescence detection platforms*. (**A**) In the one-step assay of the Cas13a/crRNA miRNA detection platform, a fluorophore (FAM)- and quencher (BHQ1)-labeled poly-U RNA reporter probe is digested by the Cas13a collateral activity, thus eliminating the fluorescence resonance energy transfer (FRET) effect and releasing fluorescence signals. (**B**) Depiction of the cascade Cas13a–Cas14a (casCRISPR) system for miRNA detection. A locked-trigger for Cas14a/sgRNA (ST-HP) functions as a bridge between Cas13a and Cas14a to serve as the substrate of Cas13a *trans*-cleavage and triggers the *trans*-cleavage activity of Cas14a. Thus, the properties of ST-HP are the most critical factor for the performance of casCRISPR. (**C**) Single-molecule miRNA quantification using droplet-digital Cas13a assay (ddCas13a). MiRNAs and a Cas13 mix are emulsified with oil into thousands of picoliter-sized droplets, each containing approximately 0 to 1 molecules of target miRNA. Once recognized by a crRNA, one target miRNA will induce cleavage of 10^4^ quenched fluorescent RNA reporters, thus yielding a fluorescently positive droplet. (ii) *Colorimetry-based approaches*. (**D**) In the Naked-Eye (NE)-CRISPR/Cas-based colorimetric assay, miRNA target-induced *trans*-ssDNA (by Cas12a/crRNA) or ssRNA (Cas13a/crRNA) cleavage triggers an aggregation behavior change for the designed AuNPs−DNA probes pair (the AuNPs−DNA probes pair loses the hybridization linkers and becomes dispersed), enabling the completion of naked-eye miRNA detection. (**E**) Schematic representation of the Cas13a-based visual detection platform vCas. In vCas, the detection results of vCas for miRNAs detection can be estimated by the naked eye, or the absorbance at 405 nm can be measured using a microplate reader. (iii) *Electrochemical detection Platforms*. (**F**) In the EM-CRISPR assay (on-chip miRNA detection), the signal obtained from the enzymatic readout (amperometric signal) reflects the amount of immobilized glucose oxidase (GOx)-conjugated and is, therefore, inversely proportional to the target concentration in the sample. Bovine serum albumin (BSA) prevents unspecific adsorption of biomolecules. (**G**) The CRISPR-Biosensor X implements different multiplexed versions of the previous electrochemical microfluidic biosensor (microfluidic multiplexed lab-on-a-chip device) by dividing its channel into subsections, creating four novel chip designs for the amplification-free and simultaneous quantification of up to eight miRNAs. (**H**) In the PECL-CRISPR chip, the electrochemiluminescence signals on a bipolar electrode (BPE) directly indicate the oxidation reaction of [Ru(phen)2dppz]2^+^-DNA complex at the anode of BPE and are proportional to target miRNA concentration, thereby enabling quantitative miRNA detection. (**I**) The COMET chip assay is composed of off-chip signal amplification (i), on-chip RNA measurements (ii), and square wave voltammetry (SWV) readout (iii). For the off-chip two-stage signal amplification, the Cas13a-mediated collateral cleavage of trigger molecules and the enzyme-free allosteric catalysis of catalytic hairpin DNA circuit (CHDC) are performed in one pot. A dual-reporter approach is employed to perform baseline drift correction for the on-chip electrochemical measurements.

Shan et al. [42] exploited the *Leptotrichia buccalis* Cas13a (LbuCas13a) protein to directly detect miRNAs with high specificity and simplicity, employing a crRNA and moderate isothermal conditions to obtain an amplified fluorescence signal, instead of relying on a cDNA synthesis step, three oligonucleotides, and different temperature cycles to replicate amplicons that are used in the stem-loop RT-PCR method. The fluorescence spectroscopy results showed that the fluorophore (FAM)- and quencher (BHQ1)-labeled poly-U RNA probe (FQ5U) introduced into the miRNA detection assay was efficiently cleaved, generating fluorescence signals [42] (Figure 2).

Thus, derived from base pairing between the crRNA and the miR-17 (chosen as the model target in this study) and the *trans*-ribonuclease activity of Cas13a, the authors detected amounts as low as 4.5 amol of miR-17 within 30 min with a dynamic range spanning four orders of magnitude from 10 amol to 100 fmol [42]. Additionally, the LbuCas13a/crRNA complex was programmed to discriminate single-nucleotide variations, even at the end of target miRNA, by rationally programmed crRNA [42].

The authors also evaluated the validity of this method by relative quantification of miR-17 in four different cell line samples, including three human breast adenocarcinoma cell lines [42]. Furthermore, quantifying miRNAs in total small RNA preparations in serum samples with high sensitivity promises a practical application of this single-step Cas13a/crRNA-based detection strategy for miRNA-related disease diagnosis [42].

### 5.2. Cascade CRISPR-Cas13 (casCRISPR) System

Recently, Sha et al. developed a sensitive and specific CRISPR-Cas-based biosensor termed casCRISPR for rapid and accurate miRNA detection, without a target amplification process (e.g., polymerase chain reaction (PCR) or recombinase polymerase amplification (RPA)), even in cell extracts and serum samples [43].

Employing this strategy, after miRNA recognition, the *trans*-cleavage activity of Cas13a/crRNA is activated. A hairpin structured DNA oligonucleotide (ST-HP or “locked-trigger”) with unpaired rU in the loop was used to explore such *trans*-ribonuclease activity. In this regard, the phosphodiester bond next to the rU of the “locked-trigger” for Cas14a/sgRNA is cleaved (Figure 2). The latter makes the “locked-trigger” transform from a stable hairpin structure into a duplex structure with a 5′-toehold, which can initiate the interaction with Cas14a/sgRNA through strand displacement [43]. Once the “locked-trigger” is cleaved, the *trans*-cleavage activity of Cas14a is elicited, resulting in efficient separation of the fluorophore (6-FAM) and the quencher (BHQ1) on the ssDNA reporter sequence, thus providing the fluorescence signals [43] (Figure 2).

Employing the *trans*-cleavage activity of Cas13a and Cas14a, the casCRISPR system can achieve miR-17 detection with a limit of detection on the order of 1.33 fM (~1000 times lower than direct Cas13a-based miRNA detection), displaying single-base specificity. Due to the Cas13a/crRNA high-fidelity recognition ability, casCRISPR could provide higher specificity for miRNA detection without the target amplification required by the qRT-PCR method, thus becoming a promising tool for miRNA diagnostics [43].

Since Cas12a also can be behave as ssDNA-activated DNase [21], the authors constructed another version of casCRISPR-coupled Cas13a with Cas12a (casCRISPR v2) comparing its performance to casCRISPR v1 (Cas13a–Cas14a) [43]. The fluorescence intensity curves obtained by these platforms demonstrated that casCRISPR v1 could detect miR-17 as low as ~6.25 fM, while casCRISPR v2 could detect ~100 fM miR-17. However, since Cas14a possesses higher cleavage efficiency than Cas12a when ssDNA is used as the activator, this could explain why Cas13a–Cas14a (casCRISPR v1) can achieve a lower detection limit and a higher signal to background ratio than Cas13a–Cas12a [43].

### 5.3. ddCas13a Assay for Direct Single-Molecule microRNA Quantification

Recently, Tian et al. described a droplet-digital Cas13a assay (ddCas13a) by harnessing the natural confinement effect, enhancing the local molecule concentration for highly efficient reaction or detection [44]. In order to potentiate the local concentration of target sequence and RNA reporter for the Cas13a system in cell-sized volumes, the authors employed a simple droplet microfluidic platform [44,45]. In this context, a mixture of target RNAs and Cas13a mixture is emulsified with oil into thousands of picoliter-sized droplet reactors. The accumulated fluorescent signal from the collateral cleavage of a single RNA target-activated Cas13a is sufficient to illuminate a picoliter-sized droplet [44,45] (Figure 2). In these conditions, after recognition by a crRNA, a single-target RNA can induce cleavage of over 10^4^ quenched fluorescent RNA reporters, thus resulting in a fluorescently positive droplet. Consequently, ddCas13a allows absolute digital quantitation (according to the Poisson distribution) of single unlabeled RNA molecules without the need for reverse transcription and amplification [44,45].

The utility of the ddCas13 assay was assessed by quantifying the miRNA-17 expression level in four different cell lines, including human breast normal cells, adenocarcinoma cells, and human glioma cells, with 100% concordance with RT-qPCR results [44]. Moreover, the applicability of this approach was also demonstrated by distinguishing the clinical serum miRNA-21 levels from breast cancer patients and healthy individuals [44].

Thus, the sensitivity, single-molecule quantitation capability, and broad applicability may render this method a promising miRNA diagnostic tool [44]. However, the developers of this platform need to continue innovating to expand its multiplexing capability and improve droplet detection.

## 6. Colorimetric miRNA Detection Based on the CRISPR-Cas13 System

### 6.1. Naked-Eye Gene Detection Platform Based on CRISPR

Colorimetric assay usually analyzes the color change exhibited by the testing solution. The naked eye or a colorimeter can monitor the result providing a quick and easy choice for miRNA detection without sophisticated instrumentation [29].

It has been demonstrated that once Cas12a/crRNA, Cas13a/crRNA, or Cas14/crRNA recognizes their target DNA or RNA, switch to the active state, wherein the single-stranded DNA (ssDNA) or RNA (ssRNA) substrates are cleaved by their collateral cleavage activity [14,21,22,24].

Yuan et al. designed and developed colorimetric detection based on gold nanoparticles (AuNPs) and DNA probes (AuNPs−DNA probes) assisted by CRISPR/Cas recognition for miRNA sensing. Through this strategy, programmable recognition of DNA by Cas12a/crRNA and RNA by Cas13a/crRNA activates the *trans*-ssDNA or -ssRNA cleavage with their respective complementary target. Target-induced *trans*-ssDNA or -ssRNA cleavage triggers an aggregation behavior change for the designed AuNPs−DNA probe pair, enabling the completion of naked-eye detection (within one hour) of different nucleic acid samples, including miRNAs [46].

This detection platform uses universal linker ssDNA or ssRNA as the *trans*-cleavage substrate for the CRISPR-Cas12a or CRISPR-Cas13a system. In addition, a pair of universal AuNPs−DNA probes was designed to hybridize to linker ssDNA or ssRNA [46]. In the absence of a target (e.g., miRNA sequences), *trans*-cleavage is not activated; thus, the linker ssDNA or ssRNA remains unbroken in the reaction. The AuNPs−DNA probe pair undergoes hybridization-induced cross-linking to form an aggregated state [46]. After Cas12a/crRNA and Cas13a/crRNA recognize their target DNA and RNA, respectively, the *trans* cleavage activity is activated, and the linker ssDNA and ssRNA are degraded. Visual detection can be estimated by determining the cross-linked and dispersed AuNPs−DNA probes [46] (Figure 2).

The sensitivity of miRNA-17 detection was as low as 500 fM, which is comparable or even superior to that of the methods based on dual-labeled fluorescent ssRNA reporter evaluated by the authors [46]. This colorimetric method had reasonable specificity for evaluating 1 nM target miRNA-17 compared to miRNA-10b, miRNA-21, and miRNA-155 at the same concentration [46]. Moreover, the CRISPR/Cas-based colorimetric assays can distinguish miRNA members with highly related sequences in the same gene family, such as miRNA-17, miRNA-20a, miRNA-20b, and miRNA-106a, with single- or double-nucleotide differences at a concentration of 1 nM [46].

### 6.2. CRISPR-Cas13a-Based Visual miRNA Detection System

Rolling circle amplification (RCA) is an isothermal enzymatic DNA replication process widely used to develop DNA, RNA, and protein sensors [47,48]. A short DNA or RNA is amplified to form a long, single-stranded DNA or RNA employing a circular DNA template and unique isothermal strand displacement DNA or RNA polymerases in an RCA reaction [47,48]. The product of RCA is a concatemer containing over 10^9^-fold tandem DNA repeats that are complementary to the circular DNA or RNA template [49].

Zhou et al. developed the CRISPR/Cas13a-based visual detection system, termed vCas, for specific and sensitive detection of miRNA [50] (Figure 2). In principle, Cas13a/crRNA collaterally cleaves the uracil ribonucleotide (rU)-bearing preprimer upon recognition of target miRNA. The remaining 5′-DNA fragment of the preprimer can initiate DNA polymerase-mediated rolling circle amplification. After its 3′-end, the above is restored by a T4 polynucleotide kinase to generate a long G-rich repeat sequence, which can form a tandem G-quadruplex to be able to act as horseradish peroxidase mimicking DNAzyme and catalyze (in the presence of hemin) the oxidation of the 2,2′-azino-bis (3-ethylbenzthiazoline-6-sulfonic acid) diammonium salt (ABTS2−) (Figure 2). As a result, the colorless mix turned green within 10 min, with which the naked eye can quantify the target miRNA [51]. The results employing this strategy indicated that vCas can provide a detection limit of 1 fM for miR-10b (a miRNA highly expressed in metastatic breast cancer cells [52,53]) with single-base specificity and can be used to analyze miRNA in serum and cell extracts [50].

The corresponding absorption spectroscopy results showed that the vCas system can reach a LOD of 1 fM for miR-10b with good linearity from 1 fM to 10 pM, which are four orders of magnitude lower than that of the one-step Cas13a detection method [50]. In addition, due to the ability of the vCas system to discriminate at a single-base level, this platform significantly distinguished highly homologous let-7 family members (abundantly expressed in the brain, regulating cell differentiation and brain tumor growth [54]) that only have a single-base difference [50].

The applicability of vCas for detecting miR-10b was evaluated in total small RNA (1.6 ng) extracted from four human cancer cell lines and in human peripheral blood serum. The detection results showed that all four cancer cells displayed varying degrees of overexpression of miR-10b compared with a cell line used as a control [50].

## 7. Electrochemical miRNA Detection Based on CRISPR-Cas13

### 7.1. Electrochemical CRISPR/CHDC Assay for miRNA-21

Electrochemical biosensors are becoming a powerful detection tool due to their excellent selectivity, sensitivity, and inexpensive instrumentation [55].

Cui et al. developed an ultrasensitive electrochemical biosensing platform for miRNA-21 determination based on the combination of the CRISPR-Cas13a system and C the catalytic hairpin assembly (CHA) reaction [56]. CHA is an enzyme-free, high-efficiency, and isothermal amplification method that can be adapted to multiple analytical formats, including fluorescent [57], electrochemical [58], colorimetric [59], surface plasmon resonance [60], and electrophoretic approaches [61]. A CHA reaction is performed using two complementary DNA strands prepared as stable hairpin structures. As a result, spontaneous interaction between the two DNA strands is kinetically blocked, as the complementary domains are locked up within the hairpin stems [36]. Single-stranded analytes initiate a typical CHA reaction, and substrate hairpins are successively opened, resulting in thermodynamically stable double-stranded structures. Under certain conditions, the CHA reaction can even achieve 600,000-fold signal amplification [36].

The CRISPR/CHDC assay employs the hairpin DNA 1 probe, which is first assembled on the surface of a gold (Au) electrode through a Au-S bond and then blocked with 6-mercapto-1-hexanol to form the biosensing electrode [56]. In the presence of target miRNA-21, the target miRNA can hybridize with the spacer region of the Cas13a/crRNA duplex to activate the *trans*-cleavage activity of Cas13a, leading to the cleavage of great hairpin DNA (which in turn contains an RNA sequence used as the *trans*-cleavage site) to release the secondary target DNA fragments (ST) [56]. Next, the ST strands are used as the initiator of the CHA reaction; that is, the ST strands would first hybridize with the hairpin DNA 1 probe on the surface of the Au electrode, and then the opened hairpin DNA 1 probe would hybridize with the methylene blue (MB)-labeled hairpin DNA 2 to liberate ST strands for the next cycle. Consequently, the MB tags are close to the Au electrode surface to generate amplified electrochemical signals [56].

By employing the CRISPR-Cas13a-mediated cascade signal amplification strategy, this electrochemical biosensor displayed a good linear relationship from 10 fM to 1 nM with a detection limit of 2.6 fM [56]. The biosensing platform’s application was evaluated for miRNA-21 detection. The analytical reliability was assayed in a clinical sample (human serum) diluted 10-fold and then added to different concentrations of miRNA-21 (10 fM, 0.1 pM, 10 pM, 100 pM, and 1 nM). The electrochemical recoveries were 98.2%, 101.0%, 97.3%, 100.9%, and 103.0%, respectively, demonstrating the CRISPR/CHDC assay applicability for detecting miRNA-21 in biological samples [56].

### 7.2. EM-CRISPR-Cas13a for miR-19b and miR-20a Detection

To address some limitations concerning error-prone amplification steps or the requirement of an appropriate primer design to avoid error-prone amplification steps, Bruch et al. first combined microfluidics with an electrochemical signals readout to develop a sensitive and selective diagnostic test. Subsequently, they applied CRISPR-Cas13a technology to this diagnostic test. They developed a low-cost and easy-to-use electrochemical microfluidic biosensor based on the CRISPR-Cas13 system (EM-CRISPR-Cas13a), replacing the synthetic nucleic acid amplification steps with a Cas13a driven signal amplification for the on-site detection of miRNAs [62].

Thus, for the CRISPR/Cas13a-powered miRNA detection, the surface of the immobilization area of the biosensor was functionalized by applying streptavidin to the chip inlet. By applying a vacuum to the channel inlet, all unbound biomolecules are removed [62]. For the activation of the Cas13a and the resulting collateral cleavage of the reporter RNA (reRNA), the Cas13a effector with its target-specific crRNA, the biotin and 6-FAM labeled reRNA, and the sample containing the target miRNA are mixed separately [62]. The mixture is subsequently applied to the microfluidic chip, where the cleaved and noncleaved reRNAs bind to the immobilized streptavidin (Figure 2). Following the incorporation of antifluorescein antibodies coupled to glucose oxidase (GOx), these can only bind to the uncleaved reRNAs, enabling an enzymatic reading of the assay (Figure 2). Hence, for the assay readout, a glucose solution is pumped through the microfluidic biosensor, where GOx catalyzes its substrate, producing H_2_O_2_, which is amperometrically detected in the electrochemical cell. The obtained amperometric signal is directly proportional to the amount of immobilized GOx, bound to the uncleaved reRNA, and, therefore, inversely proportional to the concentration of target miRNA in the sample [62].

Through this novel combination, the authors measured miRNA levels of the potential brain tumor markers miR-19b and miR-20a in serum samples from brain cancer patients without any nucleic acid amplification. The readout time of this EM-CRISPR-Cas13a biosensor was 9 min with a setup time of less than 4 h achieving a limit of detection of 10 pM using a measuring volume of less than 0.6 μL. Furthermore, this biosensor platform was also feasible for detecting miR-19b in serum samples of pediatric patients with brain cancer, demonstrating the ability of this electrochemical CRISPR-powered system for miRNA-based diagnostics as a low-cost, easily scalable, and target amplification-free molecular approach [62].

### 7.3. Simultaneous Quantification of miRNAs by CRISPR-Biosensor X

Examining the abundance of several components of interest in the same patient’s sample carries the development of diagnostic methods and devices for the multiplexing approach. However, the number of the available Cas protein types and the cross-reaction among different Cas13a proteins can limit the application of Cas13a in multiplex detection [63]. Hence, a multichannel microfluidic chip approach could overcome this inconvenience.

In this regard, more recently, under the same principle used for the EM-CRISPR-Cas13a system, Bruch et al. designed and implemented different multiplexed versions of their electrochemical microfluidic biosensor by dividing the channels into subsections, creating four novel chip designs for the amplification-free and simultaneous quantification of up to eight miRNAs through the novel system named CRISPR-Biosensor X. The above can be achieved from a single clinical sample using only one effector protein without changing the sensor or measurement setup [63].

### 7.4. PECL-CRISPR for miRNA Detection

Electrochemiluminescence (ECL) is an electrochemistry-triggered chemiluminescence technique that harnesses a low background noise and wide dynamic range, the main advantages of chemiluminescence and electrochemistry [64,65].

Zhou et al. developed an elegant platform termed CRISPR-Cas13a powered portable ECL chip (PECL-CRISPR), which utilizes the *trans* cleavage activity of CRISPR-Cas13a to mediate the exponential amplification of the ultrasensitive and high specific detection of miRNA with clear RNA recognition at a single-base resolution [66]. In principle, the assembled Cas13a/crRNA system specifically recognizes and cleaves target miRNA, thereby triggering its *trans* cleavage activity with what is possible to initiate the polymerization reaction. Through the nicking and strand extension/displacement cycle, large amounts of dsDNA products can be generated. A “light switch” [Ru(phen)2dppz]2^+^ can interact with the dsDNA products and the luminescence signal can be significantly increased due to the planar phenazine ligand of [Ru(phen)2dppz]2^+^ interacting with the base pairs in the major groove of dsDNA. The ECL signal on a bipolar electrode (BPE) directly indicates the oxidation reaction of [Ru(phen)2dppz]2+-DNA complex at the anode of BPE and is proportional to the target miRNA concentration, thereby enabling quantitative miRNA detection (Figure 2). [66]

The application ability of PECL-CRISPR has been investigated by detecting miR-17 from different human tumor cells (raw cell lysates), including human breast adenocarcinoma cells (MDA-MB-231 and MCF-7) and human hepatocellular liver carcinoma cell lines (HepG2), demonstrating that the platform holds excellent potential for miRNA diagnosis [66].

Moreover, through rationally designing the crRNA, the platform proved to be able to distinguish miR-17 and let-7 highly homologous family members by flexibly introducing a mismatch at the specific site of crRNA, regardless of the single different base locates at the 5′ or 3′ end of the target (miR-17 vs. miR-106a, let-7b vs. let-7c). The limit-of-detection that this platform was able to provide was 1 × 10^−15^ M for miR-17 (~3 orders of magnitude lower compared with the direct detection of miR-17 by LbuCas13a (LOD 1 × 10^−12^ M)) [42].

### 7.5. Cas-CHDC-Powered Electrochemical RNA Sensing Technology (COMET)

Sheng et al. developed the Cas-CHDC-powered electrochemical RNA sensing technology (COMET), which comprises a two-stage signal amplification system, through the CRISPR/Cas13a system and a catalytic hairpin DNA circuit (CHDC), integrated on a reusable electrochemical biosensor, for high-sensitivity sequential measurements of six non-small-cell lung carcinoma (NSCLC)-related RNAs, including miR-17, miR-155, miR-19b, and miR-210. For the CRISPR/Cas13a system and catalyzed hairpin DNA circuit (Cas-CHDC)-powered RNA detection, all components are thoroughly mixed in a standard reaction vessel and incubated at 37 °C, allowing a dual signal amplification [67].

COMET enables RNA detection with a broad dynamic range of eight orders of magnitude (50 aM to 5 nM) within a readout time of 6 min and an overall process time of 36 min, in a measuring volume of 10 μL. The reusable biosensor platform could selectively and sensitively identify low expression RNA targets in human serum, distinguishing early-stage patients (*n* = 20) suffering from NSCLC from healthy subjects (*n* = 30) and patients with benign lung disease (*n* = 12). The latter demonstrates the ability of the electrochemical CRISPR/CHDC system to be a fast and highly accurate tool for early cancer diagnostics [67].

Subsequently, the authors integrated amplification by Cas-CHDC into a reusable electrochemical biosensing technology on a chip to realize rapid and highly sensitive RNA detection on a miniaturized point-of-care testing device [67]. Thus, miR-17 belongs to the miR-17/92 family of groups whose members of the same family often differ by only 1–2 bases. In this regard, the COMET chip was able to distinguish miR-17 from miR-106a (1-base mismatch) and miR-20a from miR-20b (two-base mismatches) [67]. Moreover, the COMET chip’s ability to detect low abundance target RNAs in complex samples was evaluated by performing a mixture of miR-17 with miR-155 and TTF-1 mRNA at a 1:1000 M ratio (50 fM of miR-17 and 50 pM of non-target RNAs) [67]. The authors found that the COMET chip could detect miR-17 in the range of zeptomole and as low as 0.1% of background RNA mixture, which is critical for future clinical application due to the high expression of non-target RNAs in the patient’s bloodstream [67]. It is important to point out that the total cost of the chip and employed reagents can be as low as USD 0.27 per test [67].

In Table 2, the CRISPR-Cas13 based biosensors for miRNA detection are summarized.

## 8. Conclusions

MiRNAs are becoming the biomarkers in clinical diagnostics of multiple diseases due to their dysregulation associated with many different conditions, such as cancer [68,69], dementia [70], and cardiovascular diseases [71,72]. Thus, miRNA detection can be crucial for an early accurate initial diagnosis, evaluating responses to effective treatment, and improving the patient’s survival probabilities.

Designing a strategy that could broadly target and detect miRNA sequences (particularly with low expression levels) would be an invaluable resource. In this regard, in diagnostics, synthetic biology employs forward-engineering approaches that are typically focused on building sensors coupled to a measurable output to create new molecular functions [73].

Owing to the inherent advantages of colorimetric and electrochemical transduction methods and their excellent compatibility with the CRISPR-Cas13 technology, the detection of miRNAs through these novel biosensors can potentially be scaled in high-performance devices at low cost with a simple miniaturized readout [37].

It should be noted that the detection limit of CRISPR-Cas-mediated amplification-free nucleic acid detection is usually at the pM level [62], and recently, several CRISPR-Cas-based biosensors have improved the sensitivity to aM, or even zM, by target amplification [74,75]. In this respect, all the biosensors described here give us rapid, real-time, precise, and authentic information about miRNA molecule detection with desirable sensitivity and specificity. Importantly, these approaches are cheaper and cost-effective in sample and reagent consumption than the current gold standard RT-qPCR for miRNA detection.

The versatility is unprecedented for a single protein. Moreover, simply by changing the spacer sequence of crRNAs, it is possible to detect any miRNA sequence, indicating the excellent versatility of these proposed biosensing platforms. In recent years, this programmable nature of the CRISPR-Cas13 system has allowed the scientific community to move remarkably fast to develop an innovative diagnosis for a wide variety of human miRNAs, with important implications in the clinic.

However, the existence of off-target activity is not surprising, as molecular interactions are never perfectly specific [76]. Thus, the possible limitations of the CRISPR-Cas13 system could include: (1) RNA recognition of small RNA target sequences (< 22 nt) because crRNAs need to be long enough for binding [12]; (2) As the fidelity of the CRISPR-Cas13 system is directly related to the tolerance of RNA mismatches, a higher mismatch tolerance of Cas13 effectors could propitiate off-target activity [77]; (3) Added to this, the requirement of specific PFS for Cas13 effectors may be hard to satisfy; and (4) RNA segments derived from effective ssRNA cleavage mediated by Cas13 could be toxic in eukaryotic cells [12,78]. Therefore, further detailed structural studies and functional validation of Cas13 effectors in cells will be vital for defining their mechanistic differences and operational efficiency [79].

Nevertheless, although we have not yet exploited the full potential of CRISPR-Cas13-based platforms for miRNA detection and monitoring in clinical settings, this technology represents a revolutionary advancement in engineering sensors as minimalist strategies.

## Figures and Tables

**Table 1:** Comparison of the main features of Cas12, Cas13, and Cas13 protein effectors.

	Cas Effector		
Feature	Cas12	Cas13	Cas14
Size (a.a)	~800–1300 a.a	~930–1250 a.a	~530–700 a.a.
Protein domains	RuvC, NUC (Cas12a)	2× HEPN	RuvC
sgRNA length (nt)	~42–44 nt	~ 64–66-nt	~140 nt
Targeted nucleic acid	ssDNA dsDNA	ssRNA	ssDNA dsDNA
Targeted nucleic acid noncanonical *trans*-cleavage activity	ssDNA	ssRNA	ssDNA
Target substrate preference (specificity for the cleavage)	T-rich PAM [23]	PFS (3′, non-G) [12] No PFS required for Cas13d	None [22]
Accuracy	Effectively discriminates between targets at single-nucleotide resolution (dsDNA)	Can discriminate between targets at single-nucleotide resolution (ssRNA)	Effectively discriminates between targets at single-nucleotide resolution (ssDNA)
Applications	(a) Gene editing [21], (b) Nucleic acid detection [24]	(a) RNA knockdown [19], (b) RNA editing [25], (c) RNA imaging and tracking (dCas13a) [19], (d) Regulation of gene expression, (e) Nucleic acid detection [26], (f) Resistance against RNA viruses [27]	(a) DNA SNP genotyping [22], (b) Nucleic acid detection [22].

a.a., Amino acids; nt, Nucleotides; PAM, Protospacer adjacent motif; PFS; Protospacer-flanking site; ssDNA, single-stranded DNA; dsDNA, double-stranded DNA; ssRNA, single-stranded RNA; SNP, single-nucleotide polymorphism.

**Table 2:** Comparison of the CRISPR/Cas13-based biosensors for microRNA detection.

Platform	Protein Effector	Readout Techniques	Amplification Step	Limit of Detection *	miRNA Target	Refs.
Single-step assay	*Lbu*Cas13a	Fluorescence	-	4.5 attomol	miR-17, miR-10b, miR-21, miR-155	[42]
casCRISPR	*Lbu*Cas13a	Fluorescence	-	1.33 femtomolar	miR-17	[43]
ddCas13a assay	*Lbu*Cas13a	Fluorescence	-	3 attomolar (~2 copies/μL)	miR-17	[44]
Naked-Eye-CRISPR	*As*Cas12a/ *Lbu*Cas13a	Colorimetry	RPA/PCR	500 femtomolar	miR-17	[46]
vCas	*Lbu*Cas13a	Colorimetry	RCA	1 femtomolar	miR-10b, miR-17, let-7a, let-7b, let-7c	[50]
CRISPR/CHDC assay	Cas13a	Electrochemical	CHA	2.6 femtomolar	miR-21	[56]
EM-CRISPR	*Lwa*Cas13a	Electrochemical Microfluidic	-	10 picomolar	miR-19b, miR-20a	[62]
CRISPR-Biosensor X	*Lw*Cas13a	Amperometric readout (μA cm^−2^)	-	264.14 μA cm^−2^ and 246.59 μA cm^−2^ (baseline subtracted) peaks	miRNA-19b, miRNA-20a	[63]
PECL-CRISPR	*Lbu*Cas13a	Electrochemi-luminescence	EXPAR	1 femtomolar	miR-17	[66]
COMET	Cas13a	Electrochemical	-	50 attomolar	miR-17, miR-155, miR-19b, miR-210	[67]

*Lbu*Cas13a: *Leptotrichia buccalis* Cas13a; *Lwa*Cas13a: *Leptotrichia wadei* Cas13a; *As*Cas12a: *Acidaminococcus sp* Cas12a; CHDC: Catalytic hairpin DNA circuit; RPA: Recombinase polymerase amplification; PCR: Polymerase chain reaction; RCA: Rolling circle amplification; CHA: Catalytic hairpin assembly; EXPAR: Isothermal exponential amplification; 1 attomol (10^−18^ mol); 1femtomolar (1× 10^−15^ molar); 1 picomolar (1 × 10^−18^ molar); * According to Zhou et al., the limit of detection can be defined as the target concentration that yields a net signal equivalent to three times the standard deviation of three replicates of the control sample without the target [66].

## Data Availability

Not applicable.
